# Cell-Free DNA: Unveiling the Future of Cancer Diagnostics and Monitoring

**DOI:** 10.3390/cancers16030662

**Published:** 2024-02-04

**Authors:** Edoardo Francini, Pier Vitale Nuzzo, Giuseppe Nicolò Fanelli

**Affiliations:** 1 Department of Experimental and Clinical Medicine, University of Florence, 50134 Florence, Italy; 2 Department of Pathology and Laboratory Medicine, Weill Cornell Medicine, New York, NY 10065, USA; pvn4001@med.cornell.edu; 3 Division of Pathology, Department of Translational Research and New Technologies in Medicine and Surgery, University of Pisa, 56126 Pisa, Italy; nicolo.fanelli@unipi.it

As we conclude this Special Issue of 21 published articles dedicated to cell-free DNA (cfDNA) as a prognostic and predictive biomarker in solid cancers, we find ourselves gazing at a vibrant landscape of research on cfDNA. In recent years, we have witnessed a growing body of data regarding cfDNA, unveiling its outstanding potential to reshape cancer diagnostics and monitoring. This Special Issue has delved into this burgeoning field, addressing critical gaps in the knowledge and illuminating promising avenues for future exploration.

In recent years, cfDNA analysis has evolved from a blurred hypothesis to a tangible and reliable clinical tool. Its non-invasive nature, accessibility, and dynamic reflection of tumor evolution offer a stark contrast to traditional tissue biopsies [1,2,3]. Several pre-clinical and clinical studies have explored the use of cfDNA as an early detector of cancer. Yet, robust evidence is still lacking. This Special Issue further contributes to this growing body of data, showing that cfDNA methylation signatures hold promise for identifying precancerous lesions and early-stage tumors for hepatocellular carcinoma and colorectal cancer [4,5,6,7]. Beyond early detection, cfDNA has shown its utility as a predictive biomarker for certain tumors, enabling clinicians to tailor treatment strategies to individual patients based on their unique genomic profiles. In this Special Issue, cfDNA mutations were found to predict resistance to palbociclib for metastatic breast cancer, while liquid biopsies unveiled actionable mutations for metastatic castration-resistant prostate cancer [8,9,10,11]. This level of personalization promises to guide treatment choices, improving treatment efficacy and avoiding unnecessary side effects [12].

However, challenges remain as we strive to fully realize the clinical potential of cfDNA. Issues like assay standardization, cost optimization, and seamless integration into clinical workflows require further investigation. Future research must focus on addressing these hurdles, paving the way for the wider adoption of cfDNA technology in routine clinical practice.

Looking ahead, the future of cfDNA research is brimming with exciting hypotheses. Can cfDNA analysis accurately predict which patients are most likely to benefit from immunotherapy? Can it predict with great sensitivity minimal residual disease, informing preemptive intervention and preventing disease recurrence? Can the analysis of circulating tumor DNA clones unlock the secrets of tumor heterogeneity offering a deeper understanding of cancer evolution and informing more targeted therapeutic strategies?

As we contemplate these questions, let this Special Issue serve as a springboard for future endeavors. Its diverse tapestry of studies offers a roadmap for navigating the uncharted territories of cfDNA research. From early detection to treatment personalization, from minimal residual disease to tumor heterogeneity, the future of cancer diagnostics and monitoring lies intricately intertwined with the evolving story of cfDNA.
